# Supplementary material for: The Construction and Exploration of a Comprehensive MicroRNA Centered Regulatory Network in Foxtail Millet (Setaria italica L.)
Source: Front Plant Sci. 2022 May 6;13:848474. doi: 10.3389/fpls.2022.848474 (PMC9121102; doi:10.3389/fpls.2022.848474)
Supplement: Supplementary file 4 [file Data_Sheet_4.zip › Sit-miR396a_Seita.2G262800.1_496_TPlot.pdf]

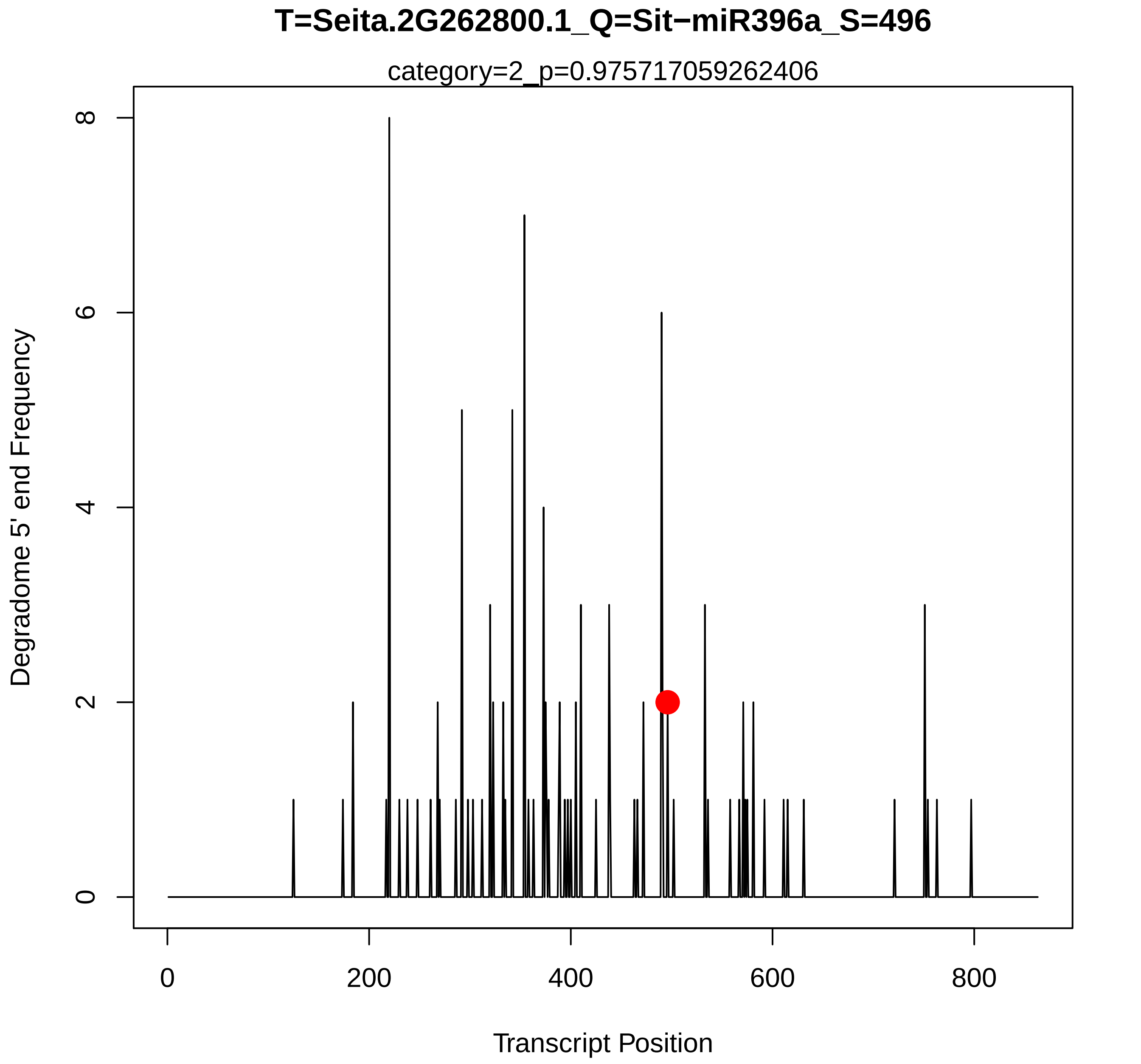
T=Seita.2G262800.1_Q=Sit−miR396a_S=496
category=2_p=0.975717059262406
Degradome 5' end Frequency
Transcript Position
0
2
4
6
8
0
200
400
600
800
